# Maternal vitamin D status in relation to cardiometabolic risk factors in children from the Norwegian Environmental Biobank

**DOI:** 10.1371/journal.pone.0318071

**Published:** 2025-02-25

**Authors:** Anna Amberntsson, Linnea Bärebring, Anna Winkvist, Lauren Lissner, Anne Lise Brantsæter, Iris Erlund, Eleni Papadopoulou, Hanna Augustin

**Affiliations:** 1 Department of Internal Medicine and Clinical Nutrition, Institute of Medicine, Sahlgrenska Academy, University of Gothenburg, Gothenburg, Sweden; 2 School of Public Health and Community Medicine, Institute of Medicine, Sahlgrenska Academy, University of Gothenburg, Gothenburg, Sweden; 3 Department of Food Safety, Division of Climate and Environmental Health, Norwegian Institute of Public Health, Oslo, Norway; 4 Centre for Sustainable Diets, Norwegian Institute of Public Health, Oslo, Norway; 5 Department of Government Services, Finnish Institute for Health and Welfare, Helsinki, Finland; 6 Institute for Nutrition and Health Research, Helsinki, Finland; 7 Division of Health Service, Global Health Cluster, Norwegian Institute of Public Health, Oslo, Norway; Johns Hopkins University Bloomberg School of Public Health, UNITED STATES OF AMERICA

## Abstract

**Background:**

Maternal 25-hydroxyvitamin D (25OHD) status has been associated with birth weight and childhood growth. Further, maternal 25OHD status may also influence cardiometabolic outcomes in childhood. This study investigated the association between maternal 25OHD concentration in pregnancy and markers of cardiometabolic risk in 7–12-year-old children.

**Methods:**

Data were obtained from the Norwegian Environmental Biobank (NEB) including 244 mother-child pairs in the Norwegian Mother, Father and Child Cohort Study (MoBa) participating in NEB part I and II. Childhood outcomes investigated were z-scores of anthropometrics, blood lipids and hormones. Associations between maternal 25OHD and individual cardiometabolic risk factors in children were assessed by linear regression, adjusted for maternal pre-pregnancy BMI, maternal education, child’s sex, age and BMI, and tested for interaction with pre-pregnancy BMI.

**Results:**

Per 10 nmol/L increase in maternal 25OHD, childhood adiponectin z-score increased by 0.067 standard deviations (p = 0.039). There were no associations between maternal 25OHD concentration and any other cardiometabolic risk factor in childhood.

**Conclusion:**

The results indicate that higher maternal vitamin D status during pregnancy may be related to higher childhood adiponectin z-score, but not with any other cardiometabolic risk marker. Whether adiponectin could be one pathway linking vitamin D to cardiometabolic health remains to be determined.

## Introduction

Cardiovascular diseases are the leading causes of mortality globally [1]. Their development is a complex process influenced by genetic and environmental factors from pre-conception throughout life. While established cardiovascular diseases like stroke or myocardial infarction are uncommon in children, cardiometabolic risk factors can appear in childhood and persist into adulthood [2,3]. Childhood cardiometabolic risk factors include exposure to cigarette smoking [4,5], unhealthy diet [6], inadequate physical activity [1,7,8], hypertension, dyslipidaemia, hyperglycaemia, and overweight and obesity [4,6,8,9].

Given that the vitamin D status of the developing fetus depends exclusively on maternal supply, low maternal vitamin D concentrations are a significant concern for their potential effects on offspring. The Developmental Origins of Health and Disease theory posits that early life exposures, including those in utero and during early childhood, can have lasting effects on epigenetics, thereby influencing health and disease risk later in life [10]. Cardiovascular diseases are examples of adult-onset conditions that may be linked to early life exposures, such as nutritional status in utero [11]. Experimental studies indicate that vitamin D could influence adipocyte formation, suggesting a potential role for vitamin D in modulating adipose tissue inflammation [12]. Specifically, low maternal vitamin D levels, measured as 25-hydroxyvitamin D (25OHD) during pregnancy, could create a suboptimal nutritional environment for the fetus, thereby increasing the risk of cardiometabolic issues in childhood [13]. Potential pathways could be through modulations in the expression of child hormones (e.g., leptin and adiponectin) or blood lipids. Leptin and adiponectin are hormones predominantly produced by adipocytes, plays a crucial role in the regulation of energy homeostasis and body weight [14], and in the regulation of glucose levels and fatty acid breakdown [15], respectively. Additionally, inadequate maternal vitamin D during pregnancy is suggested to elevate the risk of unfavourable childhood growth patterns [16].

In a meta-analysis of randomized controlled trials in children and adolescents, vitamin D supplementation resulted in increased insulin sensitivity, but also increased low-density lipoprotein (LDL) cholesterol in childhood [17]. However, no effect of vitamin D supplementation was seen on blood pressure [17,18] or blood glucose, insulin, glycated haemoglobin (HbA1c), cholesterol (total or high-density lipoprotein (HDL)), or triglycerides [17]. Observational studies have also found lower maternal 25OHD during pregnancy to be associated with higher insulin resistance at 5-10 years of age [19,20] blood pressure at 5 years of age [21], apolipoprotein (Apo) B and blood pressure at 10 years of age [22], body mass index (BMI) in infancy [16], and waist circumference at 4 and 6 years of age [23], but not associated with triglycerides, glucose, insulin [22], waist-to-height ratio, total cholesterol, LDL or HDL cholesterol, or triglycerides [20].

Studies investigating the associations between maternal 25OHD concentration in pregnancy and cardiometabolic risk factors in childhood are limited and contradictory. Additionally, it remains unclear which specific cardiometabolic risk factors might mediate this association and at what age in childhood these associations become evident. Thus, the aim of this study was to investigate the relation between maternal vitamin D status (25OHD concentration) during pregnancy and markers of cardiometabolic risk in a group of Norwegian 7–12-year-old children.

## Materials and methods

### Study population

**Fig 1:**
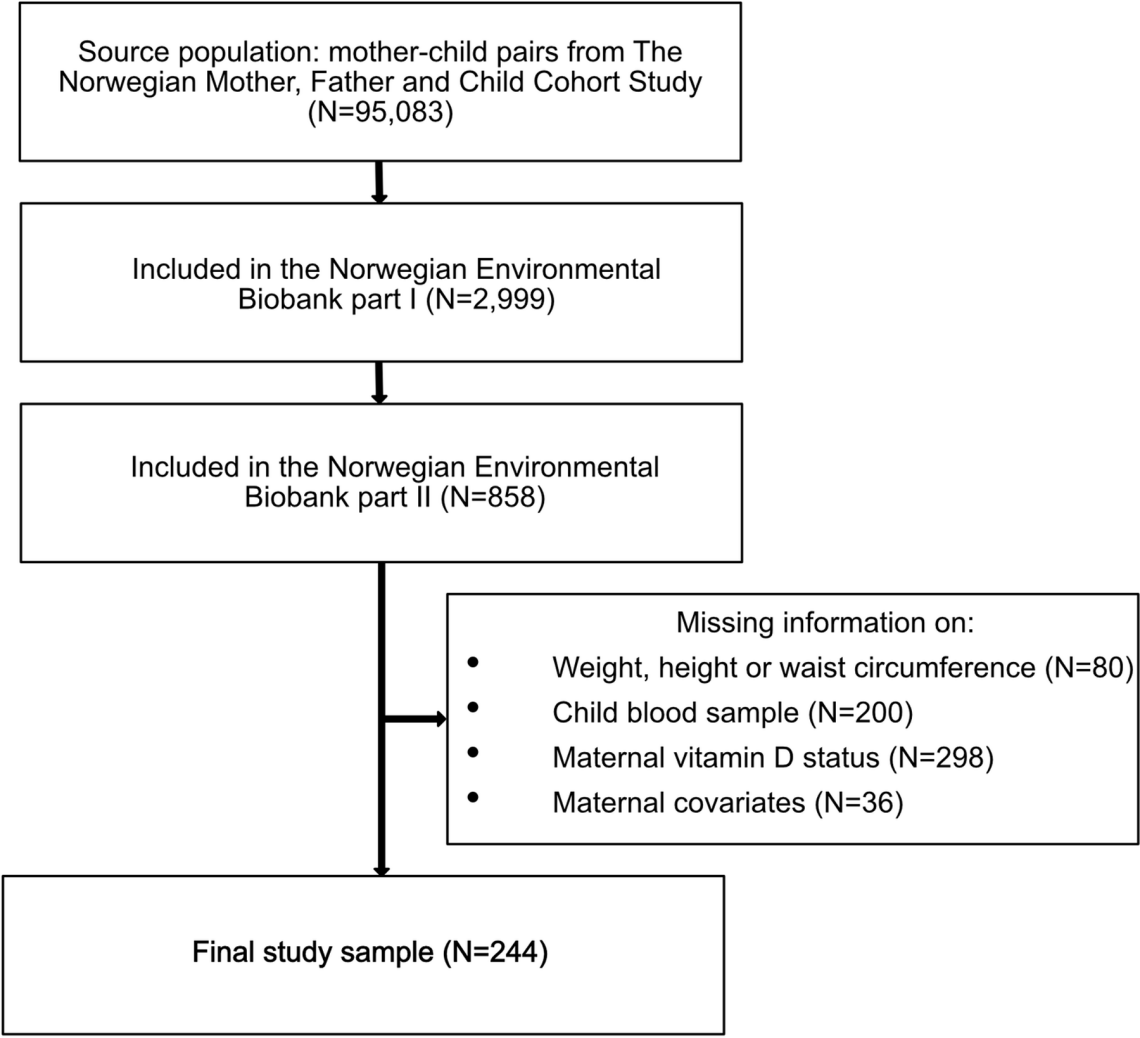
Flow chart of the study population in the Norwegian Environmental Biobank part II.

This study included mother-child pairs participating in the Norwegian Environmental Biobank part I and II, which are sub-studies of the Norwegian Mother, Father and Child Cohort Study (MoBa). A flow chart of the study sample is provided in Fig 1. MoBa is a population-based pregnancy cohort study conducted by the Norwegian Institute of Public Health [24]. The cohort includes about 114,500 children and 95,200 mothers. A subset of MoBa participants were invited to participate in The Norwegian Environmental Biobank (NEB) part I [25]. NEB part I was established with the aim of biomonitoring nutrients and environmental contaminants in MoBa participants. Inclusion criteria in NEB part I were available data from MoBa questionnaire 1–6 and father questionnaire, and available maternal plasma, urine and whole blood samples in pregnancy. The women included in NEB part I had additional elements analysed, such as 25OHD, in the blood samples drawn during pregnancy in MoBa. These women were the eligible study population for the current study (N = 2,999).

In 2016, approximately 660 triads of mothers, fathers, and children were invited to participate in The Norwegian Environmental Biobank part II [26]. The sub-study included donation of urine and a non-fasting blood sample of the children and the parents responding to a questionnaire. In the blood taken from the children, biomarkers of cardiovascular health were analysed. All children who participated in NEB part II whose mother had available data on vitamin D status during pregnancy were included in the current study (N = 244).

The current study is based on version 12 of the quality-assured data files released for research in January 2019. The establishment of MoBa and initial data collection was based on a license from the Norwegian Data Protection Agency and approval from The Regional Committees for Medical and Health Research Ethics. The MoBa cohort is currently regulated by the Norwegian Health Registry Act. The current study was approved by The Regional Committees for Medical and Health Research Ethics (REC 2019/770–12172). Data were accessed on the 22nd of April 2022. The authors did not have access to information that could identify individual participants during or after data collection. All MoBa participants provided written informed consent before enrolment into the study.

### Data collection

Self-reported data regarding maternal background characteristics (such as age, education, BMI, smoking during pregnancy, and country of origin) were collected by questionnaires during pregnancy in gestational week 15 and 30. Vitamin D intake from foods and supplements during pregnancy was estimated from a food frequency questionnaire answered by the mothers in gestational week 22. More detail about the estimated nutrient intakes from foods and supplements is described elsewhere [27].

Maternal venous blood samples were drawn in mean gestational week 18.5 (standard deviation (SD) 1.3) [25] at the routine ultrasound examination offered free of charge to all pregnant women in Norway. Unrefrigerated samples were shipped by ordinary mail in a vacutainer for long-term freezing storage in -80°C at a biobank [28]. Biochemical measurements were conducted at the Finnish Institute for Health and Welfare in Helsinki, Finland. The laboratory is accredited by the Finnish Accreditation Service and fulfils the requirements of the standard SFS-EN ISO/IEC 17025:2005. Plasma concentrations of vitamin D (25OHD_2_ and 25OHD_3_, nmol/L) were determined by the Architect 25-(OH)-D assay (accredited method), a chemiluminescent microparticle immunoassay, using the Architect ci8200 system (Abbott Laboratories, Abbott Park, IL, USA). The laboratory participates regularly in the Vitamin D External Quality Assessment Scheme [29] and met the performance target set by the DEQAS advisory panel; mean bias ±  SD from the National Institute of Standards and Technology target value was 7.7% ±  7.0. Control samples had coefficient of variation between 3.7–5.5%.

Child’s sex and birth weight were retrieved from The Medical Birth Registry, which is a national health registry containing information about all births in Norway [30].

In the questionnaire answered by the parents in NEB part II, parents were asked to provide current weight and height measurement and to measure the child’s waist circumference after given instructions. BMI was calculated by dividing the child’s weight in kilograms by their height in metres squared. Childhood overweight (including obesity) was defined according to the International Obesity Task Force [31].

Families who consented to participate received tubes for donating blood and instructions on how to send samples to the biobank at the Norwegian Institute of Public Health. Venous blood samples from the children were collected at their local doctor’s office for analysis of cardiometabolic biomarkers. The blood samples were transported by postal delivery to the biobank, with an average transit time of 1.6 days. The biomarkers included blood lipids (HDL-cholesterol, LDL-cholesterol, total cholesterol, and triglycerides), and lipoproteins (Apo A1 and B, accredited methods) Lipoprotein a and hormones (leptin and adiponectin) were also measured. Whole blood, serum and plasma were aliquoted and stored at stored at –80 °C. The Finish Institute of Health and Welfare in Helsinki performed the analyses. Total cholesterol, HDL-cholesterol, triglycerides, and lipoproteins were determined using the ARCHITECT® ci8200 System (Abbott Laboratories, Abbott Park, IL). LDL-cholesterol was calculated using Friedewald formula. For standardizing the Abbott measurements, the laboratory took part in the Lipid Standardization Program organized by Centres for Disease Control and Prevention, Atlanta, USA and External Quality Assessment Schemes organized by Labquality, Helsinki, Finland. Plasma leptin was determined by using the Human Leptin Duoset ELISA (R&D Systems Europe Ltd, Abindgon, U.K) according to the manufacturer’s instruction. Plasma adiponectin was measured by using the Human Adiponectin/Acrp30 DuoSet ELISA (R&D Systems Europe Ltd, Abindgon, U.K).

### Statistical analysis

Normality was investigated visually using histograms. Leptin and lipoprotein (a) were not normally distributed and transformed using the natural logarithm.

To facilitate the comparison of individual cardiometabolic risk factors of the children with international reference values, we standardized the following risk factors based on age- and sex-specific reference values proposed by Stavnsbo et al. [32]: waist circumference, BMI, HDL cholesterol, LDL cholesterol, total cholesterol, and triglycerides. Each individual risk variable was standardized by sex using the following equation: $\text{z}-\text{score}=\frac{\left(x-\bar{x}\right)}{SD\left(\bar{x}\right)}$

where x was each child’s measured cardiometabolic risk factor, age-predicted reference values were used as the mean (x̄), and SD the standard deviation of the reference value.

Stavsbo et al. provided reference values for single cardiometabolic risk variables in children [32]. The study pooled data from 23 cohort studies conducted between 1997–2009 from Europe (Denmark, Estonia, Norway, Portugal, and Switzerland) and US, including 15,794 children between 6 and 18 years old. The reference sample consisted of 60% Caucasian children, 16.5% African children, 15.3% Mexican children, 0.9% Asian children and 6.4% with other ethnic origin or multi-ethnic.

Associations between maternal 25OHD and individual cardiometabolic risk factors were assessed by linear regression and logistic regression for binary outcomes (risk of overweight). All outcomes of cardiometabolic risk were standardized into cohort specific z-scores. All regression models were adjusted for maternal pre-pregnancy BMI (continuous), maternal education (≤12, 13–16, ≥ 17 years), child’s sex and age (continuous). Blood lipids, leptin, and adiponectin were additionally adjusted for child’s BMI (continuous). Maternal country of origin and smoking during pregnancy were also considered but could not be included due to lack of variation in the variables. The mothers were of mostly (92.5%) Norwegian origin and almost all were non-smokers during pregnancy (96%).

Significance was accepted if p < 0.05. Interaction with pre-pregnancy BMI was investigated if p < 0.20. Stata version 16 was used for the statistical analyses (Stata Corporation, College Station, Texas).

## Results

A total of 244 mother-child pairs had complete data on the relevant covariates and were included in the current study (Table 1). The children were between 7–12 years of age, and 13% of the boys and 10% of the girls were categorized as overweight.

**Table 1:** Study population characteristics of the n = 244 women and their children.

	Median (p25th–75th) or %
**Maternal characteristics**	
Age (years)	31 (28–34)
Education	
* ≤12 years*	14.3
* 13–16 years*	51.2
* ≥17 years*	34.4
BMI (kg/m^2^)	23.0 (21.2–25.2)
Country of origin	
* Norway*	92.5
* Other country*	7.5
Smoking during pregnancy (yes)	4.1
25OHD concentration (nmol/L)	52.5 (40–65)
Vitamin D intake* (µg/day)	8.5 (5.2–14.3)
**Child characteristics**	
Age	
* 7–9 years*	20.9
* 10–12 years*	79.1
Sex	
* Girl*	53.7
* Boy*	46.3
Birth weight (kg)	3.71 (3.41–4.02)
HDL cholesterol (mmol/L)	1.6 (1.5–1.9)
LDL cholesterol (mmol/L)	2.5 (2.1–3.0)
Total cholesterol (mmol/L)	4.6 (4.2–5.1)
Triglycerides (mmol/L)	0.9 (0.6–1.2)
Apo A1 (g/L)	1.52 (1.39–1.67)
Apo B (g/L)	0.79 (0.67–0.91)
Apo B:Apo A1 ratio	0.51 (0.43–0.60)
Lipoprotein (a) (g/L)	0.092 (0.049–0.276)
Leptin (µg/L)	4.7 (3.0–9.6)
Adiponectin (mg/L)	5.9 (4.8-7.8)
**Z-scores** ^§^	**Mean (95% CI)**
Waist circumference z-score (SD)	0.08 (0.17, −0.01)
BMI z-score (SD)	−0.09 (−0.01, −0.16)
HDL cholesterol z-score (SD)	0.37 (0.50, 0.24)
LDL cholesterol z-score (SD)	0.25 (0.37, 0.13)
Total cholesterol z-score (SD)	0.53 (0.65, 0.41)
Triglycerides z-score (SD)	0.56 (0.72, 0.39)

*From foods and supplements.

^§^Reference-standardized cardiometabolic risk factor z-scores [32] standardized for age and sex.

25OHD, 25-hydroxyvitamin D; Apo, apolipoprotein; BMI, body mass index; CI, confidence interval; HDL, high-density lipoprotein; LDL, low-density lipoprotein; p, percentile; SD, standard deviation.

### Maternal vitamin D status and child’s cardiometabolic risk factors

**Fig 2:**
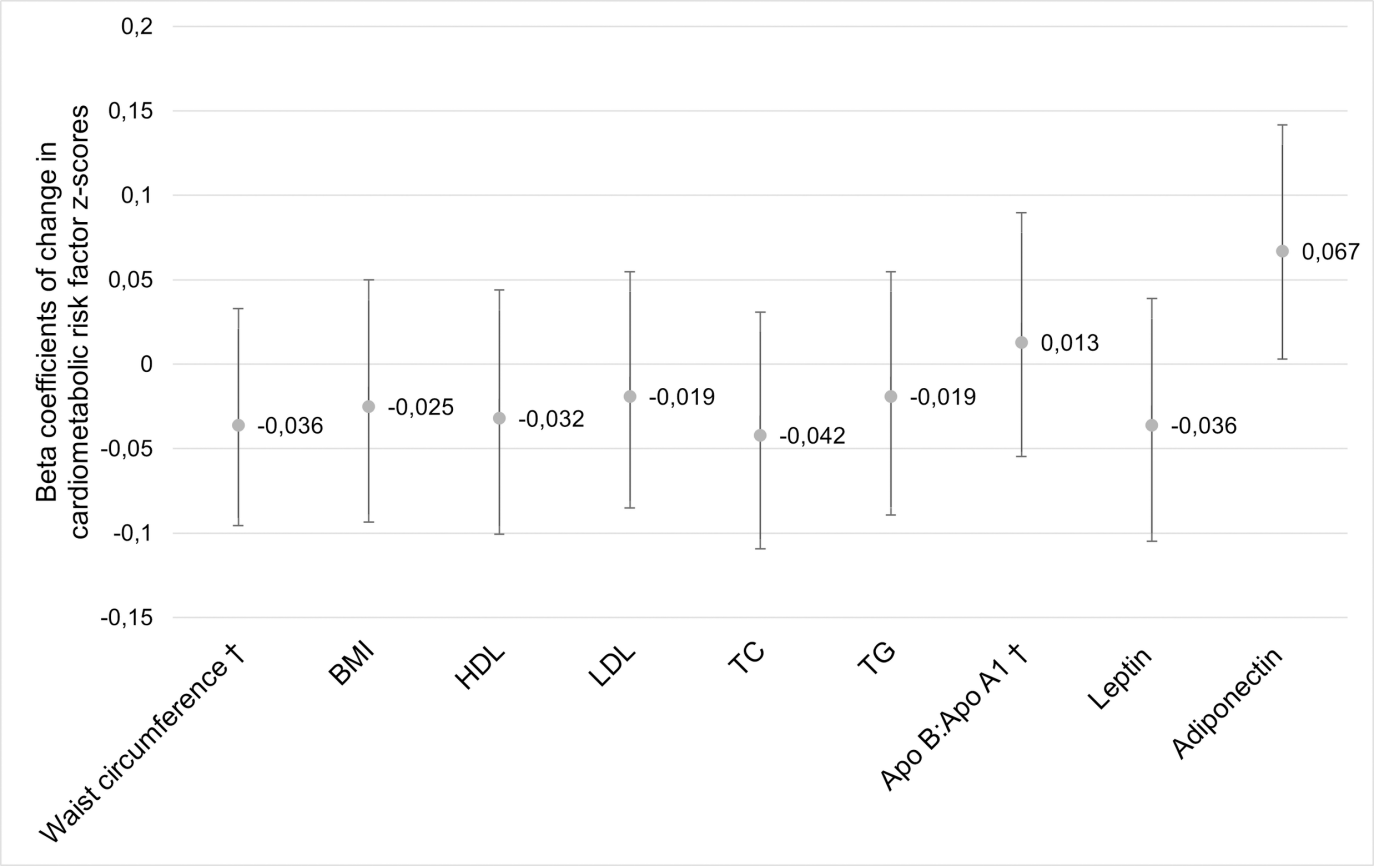
The association between maternal 25-hydroxyvitamin D (25OHD) concentration in pregnancy and childhood cardiometabolic outcome z-scores. Beta coefficients (dot) and 95% confidence intervals (whiskers) are provided per 10 nmol/L increase in 25OHD in linear regressions adjusted for maternal pre-pregnancy BMI, maternal education, child’s sex and age. Outcomes with blood lipids, leptin, and adiponectin were additionally adjusted for child’s BMI. ^†^Significant interaction (p < 0.200) with pre-pregnancy BMI. Apo, apolipoprotein; BMI, body mass index; HDL, high-density lipoprotein cholesterol; LDL, low-density lipoprotein cholesterol; TC, total cholesterol; TG, triglycerides.

Per 10 nmol/L (corresponding to approximately half an interquartile range) increase in maternal 25OHD concentration, childhood adiponectin z-score increased by 0.067 (95% confidence interval (CI): 0.0003, 0.130; p = 0.039; Fig 2 and S1 Table). Interaction with child sex in this associations was also tested but was not significant (p = 0.358). There were no associations between maternal 25OHD concentration and any other cardiometabolic risk factor, nor with the child’s risk of overweight (Odds ratio [95% CI]) (0.99 [0.96, 1.01]).

**Fig 3:**
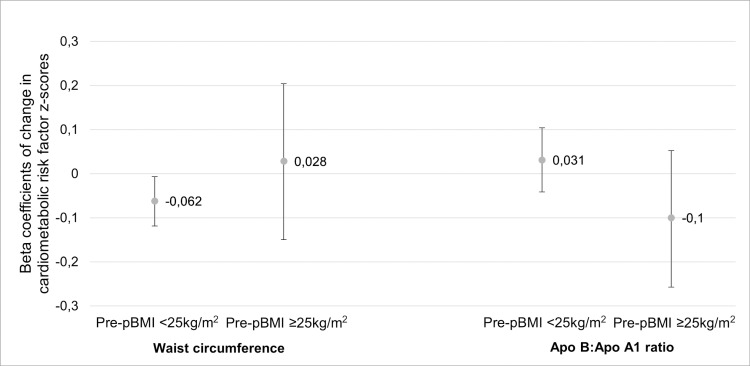
Interaction in the association between maternal 25OHD and childhood cardiometabolic outcome z-scores. Outcomes were childhood waist circumference and Apo B:Apo A1 ratio z-scores per 10 nmol/L increase in 25OHD by pre-pregnancy BMI in multivariable linear regression models. Models were adjusted for maternal education, child’s sex and age. Outcomes with blood lipids were additionally adjusted for child’s BMI. 25OHD, 25-hydroxyvitamin D; Apo, apolipoprotein; BMI, body mass index; pre-pBMI, pre-pregnancy BMI.

There was interaction with maternal pre-pregnancy BMI in the association between maternal 25OHD and childhood Apo B:Apo A1 ratio (p = 0.103) and waist circumference (p = 0.105; Fig 3 and S2 Table). In children of mothers with pre-pregnancy BMI < 25 kg/m^2^, 10 nmol/L higher maternal 25OHD and was associated with 0.062 lower childhood waist circumference z-score (95% CI: -0.118, -0.006; p = 0.032). There was no association between maternal 25OHD and childhood Apo B:Apo A1 ratio regardless of maternal pre-pregnancy BMI.

## Discussion

The results of this prospective cohort study show that higher maternal 25OHD concentration in pregnancy was significantly associated with higher childhood adiponectin z-score, but with small effect estimates (increase of 0.067 SD for every 10 nmol/L increase in maternal 25OHD concentration (p = 0.039)). Maternal 25OHD was not associated with any other childhood cardiometabolic risk factor. However, interaction analyses of pre-pregnancy maternal BMI indicated that in children of mothers with pre-pregnancy BMI in the normal range (<25 kg/m^2^), higher maternal 25OHD concentration in pregnancy was significantly associated with lower child waist circumference z-score, also with small effect estimates (-0.062 SD for every 10 nmol/L increase in maternal 25OHD concentration (p = 0.032)).

As far as we know, this is the first study to examine associations between maternal vitamin D status and concentrations of adiponectin in children. Adiponectin is positively related to insulin sensitivity in humans and predicts development of type 2 diabetes and the metabolic syndrome [33,34]. Further, adiponectin has been inversely associated with dyslipidemia and fasting glucose in children [35,36] and has been suggested to link obesity and cardiometabolic risk in childhood [35,37]. Maternal vitamin D status has previously been inversely associated with insulin resistance at 9.5 years of age [19] and at 5-6 years of age [20]. Although the effect estimates observed in these studies are small and do not reach clinical relevance, insulin resistance in childhood tends to track into adulthood [38]. In previous research, no associations have been found with fasting concentrations of glucose [20] in childhood at 5-6 years, nor with glucose or insulin at 10 or 15 years of age [22]. Although the results from previous research have shown somewhat disparate results, the observed associations in this study provide some indication that maternal 25OHD may be related to insulin resistance in childhood. Vitamin D is recognized for its role in regulating insulin secretion [39], and deficiency has been linked to the development of insulin resistance and glucose intolerance [19,40]. However, it has been suggested that the relation between vitamin D and glycaemic status is mediated by other molecules, including adiponectin [41]. A recent systematic review and meta-analysis found that vitamin D may be considered an adiponectin secretagogue, but only in subjects with diabetes [41]. The mechanism through which higher maternal vitamin D status might be related to higher childhood adiponectin and the clinical importance cannot be determined from these data and remains speculative. Whether adiponectin could be one pathway linking vitamin D to cardiometabolic health remains to be determined.

In the current study, higher maternal 25OHD concentration was associated with lower child waist circumference in children of mothers with pre-pregnancy BMI < 25 kg/m^2^. A previous study found an inverse association between maternal vitamin D status and child’s waist circumference at 4 and 6 years of age [23]. Another study found no overall association between maternal vitamin D status and the child’s waist circumference at 5 or 9.5 years of age [19]. None of the above-mentioned studies considered interaction by maternal pre-pregnancy BMI and thus, are not fully comparable with our results. However, several studies have found an inverse association between maternal vitamin D status and other measures of childhood fat mass [20,42,43]. Further, we and others have previously reported maternal pre-pregnancy BMI as an effect modifier in the association between maternal vitamin D status during pregnancy and markers of childhood adiposity [20,44,45]. Pre-pregnancy BMI is the main determinant of BMI during pregnancy and maternal BMI during pregnancy may expose the fetus to a potentially suboptimal or adverse metabolic environment, with pathophysiological consequences for a variety of organs and systems of the child [46]. The biological mechanisms underlying the potential link between vitamin D levels during pregnancy and postnatal adiposity is suggested to be caused by epigenetic changes of the fetus exposed to suboptimal nutritional environment in utero, suggestively by changes in the fetal lipolysis [47], glucose homeostasis [48], and inflammation [49].Sufficient maternal vitamin D levels may contribute to improved metabolic function in the child’s energy homeostasis by influencing adiponectin’s role in glucose production and fatty acid oxidation. While there is scarce evidence to support an association between maternal vitamin D status and childhood waist circumference, maternal vitamin D status might play a minor role in childhood body composition. However, the current biomarker status of the sampled children may be significantly influenced by both postnatal environmental factors and the prevailing food environment. Notably, the immediate environment could play a more critical role in determining adiponectin levels compared to the maternal vitamin D status during pregnancy.

The children had statistically lower BMI and higher LDL, HDL, total cholesterol and triglycerides in comparison to the international reference population [32]. These results are in line with previous results from a Norwegian population of 10-year-old children [50]. The observed disparities between the Norwegian children and the reference population could potentially be attributed to time trends in cardiometabolic risk factor levels among children and that the reference values are derived from combined data encompassing both European and American datasets [32]. The children in the current study were born between 2003-2008, while the cohorts included in the reference population were conducted between 1997-2009, including children 6-18 years of age. Further, child’s waist circumference and BMI were self-reported and measured by the parents in the current study, and blood samples in our study was not drawn fasting, which also may contribute to the observed differences in risk factors between the Norwegian children and the reference population. Additionally, selection bias may be present in the current cohort, and families with more risk factors may have been more prone to provide blood sample.

Major strengths of the study are the prospectively, objectively measured data on cardiometabolic risk factors measured in a group of healthy children, and prior measurements of maternal 25OHD concentration during pregnancy. Another strength of the present study was the use of international reference values to standardize the cardiometabolic risk factors, which allows for direct comparisons to the reference material of international cardiometabolic risk values. Limitations include a small study sample and that a minor part of the data (weight, height, and waist circumference) was reported by the parents. Exposure misclassification cannot be ruled out entirely, however, it was previously shown that serum 25OHD concentrations measured from a Finnish population study by this immunoassay was only modestly changed by standardization by Vitamin D Standardization Program protocol [51]. Further, our results originate from a specific group of mother-child pairs with good compliance to the MoBa study protocol and with a high proportion with high educational attainment. Thus, generalization to the general population should be done with caution. Finally, although we performed adjustment for potential maternal and childhood confounders, residual confounding for the observed associations might be present. In addition, the blood samples were sent unrefrigerated by ordinary mail in vacutainers for long-term freezing storage in -80°C at a biobank. This might have had some influence on sample quality, although the stability of most of these biomarkers has been shown to be relatively good [52].

## Conclusions

The results indicate that higher maternal vitamin D status during pregnancy may be related to higher childhood adiponectin z-score, but not with any other cardiometabolic risk marker. Whether adiponectin could be one pathway linking vitamin D to cardiometabolic health remains to be determined.

## Supporting information

S1 Table The association between maternal 25-hydroxyvitamin D (25OHD) concentration in pregnancy and childhood cardiometabolic outcome z-scores per 10 nmol/L increase in 25OHD, testing for interaction with pre-pregnancy BMI. (DOCX)

S2 Table The association between maternal 25-hydroxyvitamin D (25OHD) concentration in pregnancy and childhood waist circumference and Apo B:Apo A1 ratio z-scores per 10 nmol/L increase in 25OHD by pre-pregnancy BMI. (DOCX)

## Abbreviations

25OHD: 25-hydroxyvitamin D
Apo: apolipoprotein
BMI: body mass index
CI: confidence interval
HbA1c: glycated hemoglobin
HDL: high-density lipoprotein cholesterol
LDL: low-density lipoprotein cholesterol
MoBa: The Norwegian Mother, Father and Child Cohort Study
NEB: The Norwegian Environmental Biobank
OR: odds ratio
TC: total cholesterol
TG: triglycerides.
