# Supplementary material for: Maternal vitamin D status in relation to cardiometabolic risk factors in children from the Norwegian Environmental Biobank
Source: PLoS One. 2025 Feb 25;20(2):e0318071. doi: 10.1371/journal.pone.0318071 (PMC11856322; doi:10.1371/journal.pone.0318071)
Supplement: S1 Table — (DOCX) [file pone.0318071.s001.docx]

**Maternal vitamin D status in relation to cardiometabolic risk factors in children from the Norwegian Environmental Biobank**

Anna Amberntsson*^1^, Linnea Bärebring^1^, Anna Winkvist^1^, Lauren Lissner^2^, Anne Lise Brantsæter^3,4^, Iris Erlund^5,6^, Eleni Papadopoulou^7¶^, Hanna Augustin^1¶^

1. Department of Internal Medicine and Clinical Nutrition, Institute of Medicine, Sahlgrenska Academy, University of Gothenburg, Gothenburg, Sweden

2. School of Public Health and Community Medicine, Institute of Medicine, Sahlgrenska Academy, University of Gothenburg, Gothenburg, Sweden

3. Department of Food Safety, Division of Climate and Environmental Health, Norwegian Institute of Public Health, Oslo, Norway

4. Centre for Sustainable Diets, Norwegian Institute of Public Health, Oslo, Norway.

5. Department of Government Services, Finnish Institute for Health and Welfare, Helsinki, Finland.

6. Institute for Nutrition and Health Research, Helsinki, Finland.

7. Division of Health Service, Global Health Cluster, Norwegian Institute of Public Health, Oslo, Norway.

* Corresponding author

E-mail: [anna.amberntsson@gu.se](mailto:anna.amberntsson@gu.se) (AA)

¶ Shared last authorship.

S1 Table. Multivariable linear regression models of the association between maternal 25-hydroxyvitamin D (25OHD) concentration in pregnancy and childhood cardiometabolic outcome z-scores per 10 nmol/L increase in 25OHD, testing for interaction with pre-pregnancy BMI.

| Z-scores | β | 95% CI | P-value |
| --- | --- | --- | --- |
| Adiposity |  |  |  |
| *Waist circumference* | -0.036 † | -0.091, 0.021 | 0.213 |
| *BMI* | -0.025 | -0.089, 0.038 | 0.444 |
| Lipid profile |  |  |  |
| *HDL cholesterol* | -0.032 | -0.096, 0.032 | 0.316 |
| *LDL cholesterol* | -0.019 | -0.081, 0.043 | 0.542 |
| *Total cholesterol* | -0.042 | -0.104, 0.019 | 0.180 |
| *Triglycerides* | -0.019 | -0.085, 0.043 | 0.555 |
| *Apo B:Apo A1 ratio* | 0.013 † | -0.052, 0.078 | 0.688 |
| Hormones |  |  |  |
| *Leptin* | -0.036 | -0.100, 0.027 | 0.262 |
| *Adiponectin* | 0.067 | 0.001, 0.130 | 0.039 |

Abbreviations: 25OHD, 25-hydroxyvitamin D; Apo, Apolipoprotein; BMI, body mass index; HDL, high-density lipoprotein; LDL, low-density lipoprotein
All models were adjusted for maternal pre-pregnancy BMI, maternal education, child´s sex and age. Outcomes with blood lipids and hormones were additionally adjusted for child´s BMI.

† Significant interaction (p<0.200) with pre-pregnancy BMI
