# Supplementary material for: Maternal vitamin D status in relation to cardiometabolic risk factors in children from the Norwegian Environmental Biobank
Source: PLoS One. 2025 Feb 25;20(2):e0318071. doi: 10.1371/journal.pone.0318071 (PMC11856322; doi:10.1371/journal.pone.0318071)
Supplement: S2 Table — (DOCX) [file pone.0318071.s002.docx]

**Maternal vitamin D status in relation to cardiometabolic risk factors in children from the Norwegian Environmental Biobank**

Anna Amberntsson*^1^, Linnea Bärebring^1^, Anna Winkvist^1^, Lauren Lissner^2^, Anne Lise Brantsæter^3,4^, Iris Erlund^5,6^, Eleni Papadopoulou^7¶^, Hanna Augustin^1¶^

1. Department of Internal Medicine and Clinical Nutrition, Institute of Medicine, Sahlgrenska Academy, University of Gothenburg, Gothenburg, Sweden

2. School of Public Health and Community Medicine, Institute of Medicine, Sahlgrenska Academy, University of Gothenburg, Gothenburg, Sweden

3. Department of Food Safety, Division of Climate and Environmental Health, Norwegian Institute of Public Health, Oslo, Norway

4. Centre for Sustainable Diets, Norwegian Institute of Public Health, Oslo, Norway.

5. Department of Government Services, Finnish Institute for Health and Welfare, Helsinki, Finland.

6. Institute for Nutrition and Health Research, Helsinki, Finland.

7. Division of Health Service, Global Health Cluster, Norwegian Institute of Public Health, Oslo, Norway.

* Corresponding author

E-mail: [anna.amberntsson@gu.se](mailto:anna.amberntsson@gu.se) (AA)

¶ Shared last authorship.

**S2 Table.** Multivariable linear regression models of the association between maternal 25-hydroxyvitamin D (25OHD) concentration in pregnancy and childhood waist circumference and Apo B:Apo A1 ratio z-scores per 10 nmol/L increase in 25OHD by pre-pregnancy BMI.

| **Z-scores** | **β** | **95% CI** | **P-value** |
| --- | --- | --- | --- |
| Waist circumference |  |  |  |
| *Pre-pregnancy BMI <25 kg/m^2^* | -0.062 | -0.118, -0.006 | **0.032** |
| *Pre-pregnancy BMI ≥25 kg/m^2^* | 0.028 | -0.149, 0.204 | 0.755 |
| Apo B:Apo A1 ratio |  |  |  |
| *Pre-pregnancy BMI <25 kg/m^2^* | 0.031 | -0.041, 0.104 | 0.396 |
| *Pre-pregnancy BMI ≥25 kg/m^2^* | -0.010 | -0.257, 0.052 | 0.189 |

Abbreviations: 25OHD, 25-hydroxyvitamin D; Apo, Apolipoprotein; BMI, body mass index
Models were adjusted for maternal education, child´s sex and age. Outcomes with blood lipids were additionally adjusted for child´s BMI. *N=181 children of mothers with pre-pregnancy BMI <25 kg/m^2^ and N=63 children of mothers with pre-pregnancy BMI ≥25 kg/m^2^.
